# Associations between initiating antihypertensive regimens on stage I–III colorectal cancer outcomes: A Medicare SEER cohort analysis

**DOI:** 10.1002/cam4.4088

**Published:** 2021-06-29

**Authors:** Rajesh Balkrishnan, Raj P. Desai, Aditya Narayan, Fabian T. Camacho, Lucas E. Flausino, Roger Chammas

**Affiliations:** ^1^ Department of Public Health Sciences University of Virginia Charlottesville VA USA; ^2^ Universidade de São Paulo Instituto do Câncer do Estado de São Paulo Sao Paulo Brazil; ^3^ Center for Translational Research in Onc Universidade de Sao Paulo Faculdade de Medicina Sao Paulo Brazil

**Keywords:** antihypertensive agent, colorectal neoplasms, SEER program

## Abstract

**Purpose:**

Colorectal cancer (CRC) diagnosis is associated with high mortality in the United States and thus warrants the study of novel treatment approaches. Vascular changes are well observed in cancers and evidence indicates that antihypertensive (AH) medications may interfere with both tumor vasculature and in recruiting immune cells to the tumor microenvironment based on preclinical models. Extant literature also shows that AH medications are correlated with improved survival in some forms of cancer. Thus, this study sought to explore the impact of AH therapies on CRC outcomes.

**Patients and Methods:**

This study was a non‐interventional, retrospective analysis of patients aged 65 years and older with CRC diagnosed from January 1, 2007 to December 31st, 2012 in the Surveillance, Epidemiology, and End‐Results (SEER)‐Medicare database. The association between AH drug utilization on AJCC stage I–III CRC mortality rates in patients who underwent treatment for cancer was examined using Cox proportional hazards models.

**Results:**

The study cohort consisted of 13,982 patients diagnosed with CRC. Adjusted Cox proportional hazards regression showed that among these patients, the use of AH drug was associated with decreased cancer‐specific mortality (HR: 0.79, 95% CI: 0.75–0.83). Specifically, ACE inhibitors (hazard ratio [HR]: 0.84, 95% CI: 0.80–0.87), beta‐blockers (HR: 0.87, 95% CI: 0.84–0.91), and thiazide diuretics (HR: 0.83, 95% CI: 0.80–0.87) were found to be associated with decreased mortality. An association was also found between adherence to AH therapy and decreased cancer‐specific mortality (HR: 0.94, 95% CI: 0.90–0.98).

**Conclusion:**

Further research needs to be performed, but AH medications may present a promising, low‐cost pathway to supporting CRC treatment for stage I–III cancers.

## Graphical abstract

The use of antihypertensive agents following colorectal cancer diagnosis is associated with lower mortality (both all‐cause and cancer‐specific) in elderly Medicare patients. Among the studied classes of antihypertensives, ACE inhibitors and beta‐blockers seem to be associated with protective associations.

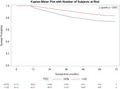

## 1 INTRODUCTION

Colorectal cancer (CRC) is the third most diagnosed cancer and the second and third leading cause of cancer deaths among men and women, respectively, in the United States. Its incidence continues to rise in developing nations.^1^ As with the majority of cancer types, surgery is the primary treatment approach, and in the case of metastasized cancers, it is preceded or followed by cytotoxic approaches such as neoadjuvant and adjuvant therapies, respectively.^2^

Solid tumor growth is associated with angiogenesis and there is a wide evidence base speaking to the relationship between cancer patient prognosis and the angiogenic potential of tumors.^3^, ^4^ However, tumor vasculature is often irregular in form due to compression by the mechanical stress induced by the proliferation of surrounding cells. Further, they have high microvascular hydrostatic pressure and transcapillary fluid movement. This, combined with poor drainage via lymphatic vessels, interstitial fibrosis, and contraction of the interstitial matrix, leads to the collection of liquid in the interstitium of solid tumors and high interstitial fluid pressure (IFP).^5^ Increases in IFP, in turn, contribute to low blood flow through the area due to high viscous resistance which has been shown to cause poor delivery of therapeutic agents to tumors and worsened outcomes.^6^, ^7^, ^8^, ^9^, ^10^ These drugs are further inhibited in their diffusion by the aforementioned increase in matrix density in solid tumors, which serves to disrupt the flow of oxygen through the microenvironment and ultimately, hypoxia. Hypoxia and uneven tumor vascularization have also been shown to be a factor contributing to the failure of cancer therapies by promoting metastases, complicating surgery, and limiting the efficacy of a variety of known cancer therapies.^11^, ^12^, ^13^, ^14^ Therefore, strategies that normalize tumor vasculature function and hypoxia to normalize the underlying tumor microenvironment may be effective for the optimization of different modalities of cancer patient management.^15^, ^16^, ^17^, ^18^, ^19^, ^20^

Hypertension is one of the most significant comorbidities encountered in patients with cancer, as evidenced by a study from Fraeman et al which found new‐onset hypertension in roughly one‐third of cancer patients.^21^ Issues may be exacerbated by chemotherapy, given that hypertension is a known risk factor for chemotherapy‐induced cardiotoxicity and has a correspondingly large influence on cancer management approaches.^22^ Of note, vascular tone, structure, and function are also altered in systemic hypertension.^23^ Common approaches to hypertension management include the application of calcium‐channel blockers, (ARB), thiazide diuretics (TD), angiotensin‐converting enzyme inhibitors (ACEI), and adrenergic β2‐receptor blockers (BB). Preference for treatment regimens is determined largely by clinical status, ethnicity, and age. Increasing evidence indicates that drugs used in the control of hypertension may also interfere with tumor vasculature function and the recruitment of immune cells to the tumor microenvironment based on preclinical models. This is effectively the reverse of the previously stated relationship between anti‐cancer drugs and hypertension. Further, elevated VEGF in patients with hypertension is correlated with cardiovascular disease risk. Given that hypertension treatment reduces VEGF levels, it may offer a novel avenue for cancer treatment with reduced risks.^24^ Unfortunately, there does not seem evidence of studies that have examined the potential associations between antihypertensive (AH) medication use on outcomes in CRC patients.

To examine whether AH medications in cancer confer protective benefits, we evaluated the impact of AH regimens used by patients with stage I–III CRC in the combined SEER‐Medicare database. We also analyzed if the use of specific classes of AH drugs would impact the efficacy of further cancer treatment and CRC‐specific mortality.

## 2 METHODS

The SEER‐Medicare data are a combination of the SEER program of cancer registries in the United States which collects clinical, demographic, and cause of death information for those with cancer. As a subset of the SEER Database, it specifically collects Medicare claims for covered health services for Medicare patients until their death. The Medicare insurance program generally accepts those who are over the age of 65, younger individuals with disabilities, or those with End‐Stage Renal Disease (kidney failure requiring dialysis or transplant). The SEER‐Medicare database analytic variables include patient demographics at diagnosis (e.g., age and gender), tumor characteristics, Medicare enrollment information, International Classification of Diseases, Ninth Revision, Clinical Modification diagnoses and procedure codes, Healthcare Common Procedure Coding System and Current Procedural Terminology codes, and prescription claims data.^25^

**FIGURE 1:**
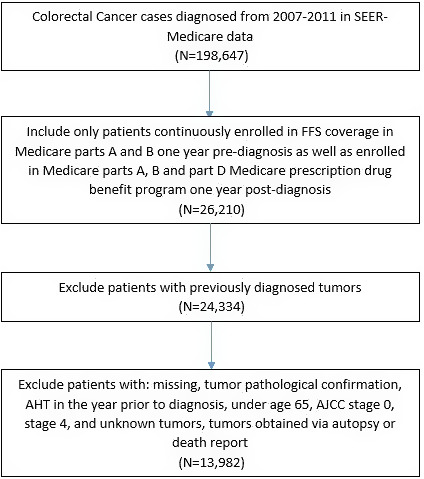
Derivation of the study population

We received Institutional Review Board approval for this analysis prior to analytic procedures. The initial study cohort included patients diagnosed with CRC from January 1, 2007, to December 31, 2011. This was defined as SEER codes (identifiers for diagnosis and/or procedures in the database) C18.0–C18.9 (malignant neoplasm of colon), C20.9 (rectum), and C19.9 (rectosigmoid junction). Inclusion criteria were first CRC diagnosis, age 65 years or older, if the diagnosis was pathologically confirmed, and whether the diagnosis was reported from autopsy or death report. Patients were included only if they were continuously enrolled in fee‐for‐service Medicare Parts A and B 12 months before diagnosis, and continuously enrolled in fee‐for‐service Medicare Parts A and B as well as Medicare Part D prescription drug benefit program at least 12 months post‐diagnosis. Patients with AH drugs prior to diagnosis, patients with AJCC stage 0 and stage IV tumors were also excluded. Patients with AH prior to diagnosis were excluded as outcomes for these patients would not be available and would not be relevant to the analysis of AH drugs on tumor progression. AJCC Stage 0 tumors were excluded as it refers to tumors that have not yet been staged and stage IV, already metastasized tumors, were excluded as this paper examined disease progression. Figure 1 shows how the study sample was derived.

The following variables were extracted for analysis from the SEER cancer registry database: age at diagnosis, year at diagnosis, sex, race (non‐Hispanic White, non‐Hispanic Black, and others), marital status, and clinical tumor characteristics including stage and tumor grade. Surgery and chemotherapy were identified from claims. Adherence was assessed by the proportion of days covered (PDC) measured. This measure has been validated in several studies.^26^ A conventional cutoff of 0.80 for PDC was used to categorize adherence (PDC  > 0.8) and non‐adherence (PDC ≤ 0.8), truncated to the range of 0 to 1. Adherence was then calculated for the patients who were continuously enrolled in the Part D prescription drug benefit program for at least 1‐year post‐initiation of AH treatment (AHT). AHT drugs included ACE inhibitors, angiotensin receptor blockers, beta‐blockers, and TD. AHT drugs were identified using NDC codes. Only new initiation of AHT was included and counted to calculate adherence. The follow‐up started 1 year after the initiation of AHT medication, and only subjects who survived up until the end of the follow‐up were included. The comorbidities were assessed using the Klabunde et al modification of the Charlson comorbidity index available from SAS macros^27^, based on physician and outpatient claims separated by at least 30 days to identify unique health conditions. Claims were searched during a 1‐year time window pre‐diagnosis date in physician, outpatient, and hospital claims, as described elsewhere.^28^ Monotherapy was defined as patients taking only one class of AHT. Cancer type was defined as patients having either colon cancer or rectal cancer. Radiation was defined as a patient receiving radiation therapy.

The primary outcome of statistical analysis was CRC‐specific (CRCS) mortality, as determined using the underlying cause of death found in SEER files. The association between AHT application and cancer‐specific mortality was modeled using Cox proportional hazards regression models to produce hazard ratios (HRs) and the associated 95% confidence intervals (CIs). Kaplan–Meier Survival Analysis was also conducted and survival was tested using the log‐rank test. The follow‐up started 1 year after diagnosis. Adherence to AHT was calculated using the PDC measure. A p value of 0.05 was considered statistically significant. The analysis was performed using SAS software (version 9.4 SAS Institute Inc., Cary, NC, US).

## 3 RESULTS

The patient population diagnosed with CRC during 2007–2011 included in this study (*n* = 13,982) was stratified based on AHT use during follow‐up. The demographic and clinical characteristics of this cohort are summarized in Table 1.

**TABLE 1:** Baseline characteristics of patients diagnosed with colorectal cancer during 2007–2012 based on antihypertensive use during follow‐up (N=13,982)

Antihypertensive Use^a^	Non‐AH users (*n* = 2553)		ACEI users^b^ (*n* = 5803)		ARB users^b^ (*n* = 1171)		BB users^b^ (*n* = 8025)		TD users^b^ (*n* = 6927)		Total (*n* = 13,982)	
	*N*	%	*N*	%	*N*	%	*N*	%	*N*	%	*N*	%
Year of diagnosis												
2007	464	18.17	1380	23.78	219	18.70	1826	22.75	1625	23.46	3003	21.48
2008	522	20.45	1269	21.87	226	19.30	1711	21.32	1533	22.13	2955	21.13
2009	485	19.00	1150	19.82	236	20.15	1576	19.64	1383	19.97	2727	19.50
2010	491	19.23	1030	17.75	246	21.01	1464	18.24	1246	17.99	2605	18.63
2011	591	23.15	974	16.78	244	20.84	1448	18.04	1140	16.46	2692	19.25
Age at diagnosis (years)												
65–69	603	23.62	1116	19.23	212	18.10	1322	16.47	1089	15.72	2568	18.37
70–74	636	24.91	1390	23.95	276	23.57	1777	22.14	1419	20.49	3209	22.95
75–79	528	20.68	1245	21.45	272	23.23	1736	21.63	1483	21.41	2992	21.40
80–84	457	17.90	1066	18.37	245	20.92	1622	20.21	1473	21.26	2728	19.51
85+	329	12.89	986	16.99	166	14.18	1568	19.54	1463	21.12	2485	17.77
Sex												
Female	1492	58.44	3289	56.68	760	64.90	4727	58.90	4289	61.92	8283	59.24
Male	1061	41.56	2514	43.32	411	35.10	3298	41.10	2638	38.08	5699	40.76
Marital status												
Single	1261	49.39	3303	56.92	612	52.26	4583	57.11	4123	59.52	7763	55.52
Married	1292	50.61	2500	43.08	559	47.74	3442	42.89	2804	40.48	6219	44.48
Race/ethnicity												
Non‐Hispanic white	2097	82.14	4673	80.53	852	72.76	6467	80.59	5642	81.45	11229	80.31
Non‐Hispanic black	164	6.42	567	9.77	86	7.34	739	9.21	683	9.86	1195	8.55
Others	292	11.44	563	9.70	233	19.90	819	10.21	602	8.69	1558	11.14
Chemotherapy	1768	69.25	4148	71.48	834	71.22	5843	72.81	5092	73.51	3993	28.56
Surgery	2438	95.50	5640	97.19	1141	97.44	7782	96.97	6694	96.64	13497	96.53
Stage at diagnosis												
I	874	34.23	1910	32.91	405	34.59	2654	33.07	2288	33.03	4640	33.19
II	875	34.27	2153	37.10	414	35.35	2939	36.62	2549	36.80	5054	36.15
III	804	31.49	1740	29.98	352	30.06	2432	30.31	2090	30.17	4288	30.67
Grade												
Well‐differentiated	225	8.81	506	8.7	95	8.1	701	8.74	623	8.99	1246	8.91
Moderately differentiated	1701	66.63	4052	69.83	823	70.28	5533	68.95	4730	68.28	9544	68.26
Poorly differentiated	401	15.71	838	14.44	179	15.29	1226	15.28	1062	15.33	2147	15.36
Undifferentiated	65	2.55	116	2.00	17	1.45	162	2.02	150	2.17	297	2.12
Unknown	161	6.31	291	5.01	57	4.87	403	5.02	362	5.23	748	5.35
Metformin use												
Yes	239	9.36	1462	25.19	273	23.31	1695	21.12	1498	21.63	2618	18.72
No	2314	90.64	4341	74.81	898	76.69	6330	78.88	5429	78.37	11364	81.28
Diabetes												
Yes	393	15.39	2268	39.08	391	33.39	2857	35.60	2586	37.33	4297	30.73
No	2160	84.61	3535	60.92	780	66.61	5168	64.40	4341	62.67	9685	69.27
CCI												
0	1604	62.83	2025	34.90	474	40.48	2833	35.30	2239	32.32	5882	42.07
1	579	22.68	1676	28.88	348	29.72	2186	27.24	1955	28.22	3822	27.34
2+	370	14.49	2102	36.22	349	29.80	3006	37.46	2733	39.45	4278	30.60
Monotherapy	–	–	945	16.28	365	31.17	1710	21.31	1238	17.87	4258	30.45
Hypertension												
Yes	975	38.19	4621	79.63	962	82.15	6337	78.97	5492	79.28	9741	69.67
No	1578	61.81	1182	20.37	209	17.85	1688	21.03	1435	20.72	4241	30.33
Cancer type												
Colon	1934	75.75	4498	77.51	956	81.64	6347	79.09	5482	79.14	10899	77.95
Rectal	619	24.25	1305	22.49	215	18.36	1678	20.91	1445	20.86	3083	22.05
Radiation												
Yes	304	11.1	596	10.7	89	7.60	754	9.40	645	9.31	1407	10.06
	220	86.6	516	88.1	1076	91.89	7184	89.52	6203	89.55	12418	88.81
Unknown	29	1.14	71	1.22	6	0.51	87	1.08	79	1.14	157	1.12

Abbreviations: ACEI, angiotensin‐converting enzyme inhibitors; ARB, angiotensin II receptor blockers; BB, beta‐blockers; TD, thiazide diuretics.

^a^ Columns of antihypertensive medication use are not mutually exclusive. Those using combination antihypertensive medications are included in multiple columns.

^b^ Antihypertensive use during the year following diagnosis.

### 3.1 Correlates of colorectal cancer‐specific mortality

A range of factors was found to be associated with higher cancer‐specific mortality in elderly Medicare patients (Table 2). Male sex was found to be significantly associated with increased CRCS mortality (HR: 1.07, 95% CI: 1.03–1.13). Patients with a marital status of single had greater mortality for patients as well (HR: 1.08, 95% CI: 1.03–1.13). With respect to procedures, patients who did not receive chemotherapy possessed a higher mortality rate than those who did (HR: 1.07, 95% CI: 1.01–1.13). Further, not receiving surgery was found to be associated with higher mortality (HR: 1.39, 95% CI: 1.23–1.58). Tumor stage and tumor grade were not found to be significantly associated with patient mortality. In addition, as determined by the Charlson Comorbidity index, patients with a higher comorbidity index (1 or 2+ comorbidities relative to no comorbidities) were associated with a higher risk of mortality (HR for 1 comorbidity: 1.08, 95% CI: 1.02–1.14 and HR for 2+ comorbidities: 1.21, 95% CI: 1.14–1.28).^29^ Cancer type and receipt of radiation therapy were not significantly associated with patient mortality.

**TABLE 2:** Risk of mortality (cancer‐specific survival) among the users of antihypertensive medication (PDC ≥ 80% vs. PDC < 80%). (*N* = 13,982)

		**Hazard Ratio (95% CI)**
PDC	>=80%	0.94 (0.90 to 0.98)
	<80%	Reference
Age		1.01 (1.00 to 1.01)
Sex	Male	1.07 (1.03 to 1.12)
	Female	Reference
Marital status	Single/Other	1.08 (1.03 to 1.13)
	Married	Reference
Race	Black	0.97 (0.91 to 1.05)
Race	Other	1.04 (0.98 to 1.11)
	White	Reference
Chemotherapy	No	1.07 (1.01 to 1.13)
	Yes	Reference
Surgery	No	1.39 (1.23 to 1.58)
	Yes	Reference
Stage	Stage II	0.98 (0.93 to 1.03)
	Stage III	1.00 (0.95 to 1.06)
	Stage I	Reference
Tumor grade	Moderately differentiated	1.04 (0.97 to 1.11)
	Poorly differentiated	1.05 (0.96 to 1.14)
	Undifferentiated	1.30 (1.11 to 1.51)
	Well differentiated	Reference
Metformin	Yes	1.04 (0.98 to 1.10)
	No	Reference
Diabetes	Yes	1.02 (0.96 to 1.08)
	No	Reference
CCI	1	1.08 (1.02 to 1.14)
CCI	2+	1.21 (1.14 to 1.28)
	0	Reference
Monotherapy	Yes	1.15 (1.10 to 1.20)
	No	Reference
Hypertension	Yes	1.13 (1.07 to 1.18)
	No	Reference
Cancer Type	Rectal	0.99 (0.94–1.06)
	Colon	Reference
Radiation	Yes	1.01 (0.92–1.10)
	Unknown	1.18 (0.98–1.43)
	No	Reference

### 3.2 Relationship between adherence and mortality

**FIGURE 2:**
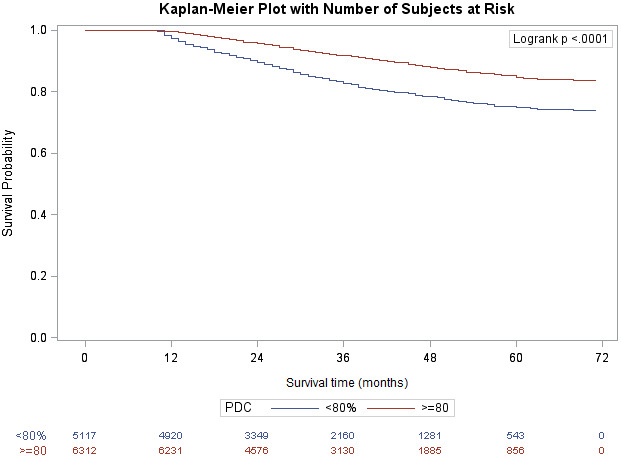
Kaplan–Meier survival curve over 6 years, by the proportion of days covered (PDC) (*N* = 11,429)

We also examined the relationship between adherence to AH medication, represented by the PDC measure (Table 2). Lower adherence was set at PDC lower than 80% and higher adherence was set as equal to or greater than 80%. A Kaplan–Meier analysis was also used to determine CRC‐specific in the groups based on adherence. There was a clear association found between increased adherence to AH medications and reduced CRCS mortality in patients starting these medications after CRC diagnosis relative to those who did not after adjusting for cancer stage and treatment (HR: 0.94, 95% CI: 0.90–0.98) (Figure 2). At follow‐up, patients in the <80% adherence group had a CRCS survival of 74% compared with 83.6% in the group with ≥80% adherence group.

### 3.3 Correlates of cancer‐specific mortality among users of antihypertensive, ACEIs, ARBs, BBs, and TDs

We subsequently focused on exploring CRCS mortality differences between patients using each of the studied AH therapeutic drugs: Overall, ACEIs, ARBs, BBs, and TDs (Table 3). Overall, AH users conferred positive associations on patient mortality. Among the different classes of drugs, it was found that ACEIs (HR: 0.84, 95% CI: 0.80–0.87), BBs (HR: 0.87, 95% CI: 0.84–0.91), and TDs (HR: 0.83, 95% CI: 0.80–0.87) also conferred positive associations on patient mortality. No association between drug class and patient mortality was found in the case of ARBs. Broadly, the use of AH medications showed a significant correlation with decreased mortality (Table 3).

**TABLE 3:** Cox proportional hazards model evaluating cancer‐specific survival for all patients diagnosed with colorectal cancer among antihypertensive and non‐hypertensive medication users (*N* = 13,982)

	Hazard^†^ Ratio (95%CI)*
Antihypertensive users	0.79 (0.75 to 0.83)
ACEI users	0.84 (0.80 to 0.87)
ARB users	0.96 (0.89 to 1.03)
BB users	0.87 (0.84 to 0.91)
TD users	0.83 (0.80 to 0.87)

^†^ Adjusted for age, sex, marital status, race and/or ethnicity, chemotherapy, surgery, stage, grade, metformin, diabetes, Charlson comorbidity index, monotherapy, hypertension, cancer type, radiation.

* ACEI, angiotensin‐converting enzyme inhibitors; ARB, angiotensin II receptor blocker; BB, beta‐blockers; CI, confidence interval; TD, thiazide diuretics.

## 4 DISCUSSION

This study examined the potential influence of AH medication initiation following CRC diagnosis based on the hypothesis that addressing hypertension, a common comorbidity of cancers, could offer protection for CRC patients. Our study evaluated the impact of these AHs from the Medicare SEER database during a contemporary time frame and found that ACEIs and TD provide the most significant benefit to the patient's survival and outcomes for those with stage I–III cancers. Our findings of the protective association between survival and the AH treatment with ACEIs are consistent with a previous study that described a significantly increased rate of pathologic complete response after neoadjuvant treatment among patients with rectal cancer.^30^ Moreover, our results show an association between increased adherence to AH medications and reduced CRCS mortality in patients starting these medications after stage I, II or III CRC diagnosis relative to those who did not. Although further analysis is necessary, this increment of survival may be associated with a higher dose exposure, as a long‐term/high‐dose exposure to ACE‐Is/ARBs was associated with a decreased incidence of CRC mortality.^31^

Several AH drugs have been recently studied in the context of cancer treatment. The angiotensin‐receptor blocker losartan has been shown to be potentially antiangiogenic in the maximally tolerated dose.^32^ Losartan may also be used in the normalization of tumor vascularization, which was indicated previously as a potential avenue for improving the distribution of oxygen and drugs, such as 5‐fluorouracil, a chemotherapeutic agent used in the management of CRCs.^33^, ^34^ Recent clinical trials seeking to apply total neoadjuvant approaches to advanced pancreatic cancer applied Losartan, a drug used to treat hypertension, in tandem with FOLFIRINOX (fluorouracil, leucovorin, oxaliplatin, and irinotecan) found evidence of downstaging of locally advanced pancreatic ductal adenocarcinoma and an R0 resection rate of 61%.^35^ Further, a phase II clinical trial is recruiting pancreatic cancer patients to assess the impact of combining chemoradiotherapy and losartan with nivolumab (immunotherapy) on survival and the proportion of patients with R0 resection.^36^ Another study is recruiting pancreatic cancer patients for a phase I clinical trial to compare the safety and efficacy of losartan plus hypo‐fractionated radiation therapy following chemotherapy.^37^ In addition, a double‐blinded, placebo‐controlled, randomized, phase III trial found that, despite the possible interference of losartan on VEGF‐mediated angiogenesis, its use did not show any impact on steroid requirements during radiotherapy to reduce peritumoral edema in newly diagnosed glioblastoma patients.^38^

Losartan has also been shown as an effective means of targeting the angiotensin signaling axis in order to reduce extracellular matrix content, thereby increasing chemotherapeutic efficacy in ovarian cancer.^39^ Aligned with this, an open‐label study is recruiting patients with glioblastoma and metastatic brain tumors from non‐small cell lung cancer to assess the dose–response relationship of losartan on imaging‐based measures of tissue perfusion and mechanical forces.^40^ Other trials are evaluating the use of losartan as neoadjuvant treatment.^41^, ^42^ Therefore, neoadjuvant therapy with losartan or others ARBs, which is relatively inexpensive and considered safe, is a promising treatment. However, the treatment’s efficacy must be further elucidated by the way of randomized clinical trials.^43^ Bevacizumab, an anti‐VEGF monoclonal antibody, has also been shown to prolong survival in ovarian and cervical cancer with chemotherapy.^44^ In addition, FOLFOXIRI plus bevacizumab has been demonstrated to improve the overall survival and progression‐free survival of CRC patients. Because of that, the treatment pathway presents a very effective first‐line regimen, regardless of ethnicity, to improve the outcome of patients with metastatic CRC.^45^ A common side effect of treatment with bevacizumab is arterial hypertension, which is easily managed by standard AH therapy. Although limited by the small sample size, a meta‐analysis reported that the occurrence of bevacizumab‐induced hypertension in patients with metastatic CRC was highly associated with improvements in progression‐free survival and overall survival.^46^ Additionally, a retrospective study of 315 patients with CRC found that patients with bevacizumab‐induced hypertension had a median overall survival of 42.6 months, while normotensive patients had 20.6 months (*p* = 0.00071).^47^ Thus, this bevacizumab‐induced hypertension might work as a prognostic predictor, once it probably has the potential to estimate the anti‐VEGF efficacy and activity of the treatment. But we should not lose sight of the possible influence that the AH drugs, used to manage this elevation of blood pressure, can have on cancer outcomes. Therefore, this correlation needs to be assessed by further research.

In this study, we found a statistically significant protective association of the use of β‐blockers on CRC‐specific mortality. Propranolol, a β2‐adrenergic receptor blocker, has been shown to interfere with NK cell homing to tumors.^48^ It has also been shown to reduce the production of angiogenic factors, such as VEGF, by macrophages and cancer cells while also downregulating the rapidly accelerated fibrosarcoma (RAF)–mitogen‐activated protein kinase (MAPK) pathway, inhibiting angiogenesis.^49^ Propranolol has been used to treat infantile hemangiomas (IH) with relative success.^50^, ^51^ Although the precise mechanisms of action of propranolol on IHs remain unclear, its angiogenic inhibition properties are one of the hypotheses for its action. According to this, propranolol and possibly other beta‐blockers may have a role in normalizing tumor vasculature and, consequently, improving the delivery of drugs to the tumor environment. Barron et al.^52^ have shown that the use of a beta‐blocker with beta‐2 receptor activity before breast cancer diagnosis can reduce breast cancer progression and mortality. R. Udumyan et al.^53^ have found that b‐adrenergic receptor blockers, particularly non‐selective types, are associated with lower liver cancer mortality in patients with primary hepatocellular carcinoma.

Haldar et al. and Shaashua et al. have evaluated the safety and short‐term efficacy of perioperative propranolol and etodolac (COX2 inhibitor) treatment on colorectal and breast cancers, respectively.^54^, ^55^ Drugs were well tolerated and the treatment significantly decreased tumor‐infiltrating CD14+ monocytes, which are associated with tumor progression and metastatic disease, and reduced markers of epithelial‐to‐mesenchymal transition (EMT), reducing the pro‐metastatic capacity of the malignant tissue. Based on this finding, a randomized phase II clinical trial is recruiting CRC patients that are going to undergo curative surgery, to assess the consequences of the treatment with propranolol and etodolac on disease‐free survival and on pro‐ and anti‐metastatic processes, through biomarkers in blood and in extracted tumor tissue.^56^ Similarly, a double‐blind placebo‐controlled two‐arm phase II clinical trial that combines a beta‐blocker with a COX2 inhibitor is recruiting primary pancreatic cancer patients to assess the efficacy and safety of this therapy.^57^ Some other clinical trials, which will help us to understand the impact of the use and effectiveness of propranolol on cancer patient management, are still ongoing. These trials include the treatment of metastatic soft tissue sarcoma, melanoma, refractory solid tumors in children and teenagers, gastric cancer, prostate cancer, and breast cancer.^58^, ^59^, ^60^, ^61^, ^62^, ^63^, ^64^

Beyond its use on neoadjuvant treatment, several clinical trials seek to assess whether AH treatment may have an action on the prevention of anthracycline‐ and trastuzumab‐induced cardiotoxicity. Although evidence is not clear, it seems that the addition of a beta‐blocker early in the treatment of cancer patients who are undergoing anthracycline or trastuzumab treatment can have beneficial associations in preserving left ventricular ejection fraction and preventing chemotherapy‐induced cardiotoxicity.^65^ A recent clinical trial, however, found that neither lisinopril nor carvedilol led to a difference in LVEF reduction in patients with HER2 breast cancer receiving trastuzumab. The same study found that both lisinopril (ACEi) and carvedilol (BB) prevented cardiotoxicity in patients with HER2‐positive breast cancer treated with anthracyclines.^66^ In contrast with this, despite a significant reduction in troponin levels and diastolic dysfunction, a prospective, randomized, double‐blind, placebo‐controlled study did not find any difference in LVEF between carvedilol‐ and placebo‐treated patients.^67^ Treatment of CRC patients with TD also showed a protective association with cancer‐specific mortality. A similar finding was described in another retrospective SEER‐Medicare cohort study, in which thiazide diuretics reduced ovarian cancer‐specific mortality (HR of 0.82, 95% CI 0.68–0.99%).^68^

Although data are data that AH drugs can interfere with tumor vasculature and microenvironment, it is hard to determine whether our results are due to the impact of the AH medication or the hypertension control. Some studies have already associated hypertension with increased cancer risks,^69^, ^70^ which might suggest that hypertension control could be an approach for cancer prevention. In patients with mild hypertension and low‐moderate cardiovascular risk, treatment often began with lifestyle changes.^71^ There are little literature data that analyzed these interventions with cancer outcomes. Some of these strategies, like the DASH diet and physical activity, were related to decreased incidence of CRC and reduced cancer mortality, though the correlation with hypertension control was not assessed.^72^, ^73^, ^74^ Therefore, it is possible that AH drugs can help achieve better outcomes on cancer patients by both mechanisms, interfering with tumor microenvironment and vasculature and also controlling blood pressure, but more research is needed to assess these correlations and potential shared mechanisms.

Despite such research, there is a sparse evidence base regarding the use of the AH drug class for cancer treatment. Thus, the results of this study are novel and suggest future research analyzing the application of AHs as a tool to improve cancer‐related mortality. In this study, we examined CRC outcomes, however, it is necessary to also investigate mortality risk in other cancers such as gastric and bladder cancer. Furthermore, we examined the application of only the four major classes of AH agents, leaving room for further study of substances such as metformin for their potential onco‐protective associations. Research collaborations exploring this space are necessary to discover novel treatment approaches for cancers.

### 4.1 Limitations

There are several limitations to our study. First, this study only examined data spanning 5 years, and was thereby limited by time span as well its retrospective nature and inherent biases. Second, the use of prescription Part D claims for adherence estimation may not represent patient medication consumption data accurately as it does not speak to patient behaviors. Third, a limitation intrinsic to administrative claims database analysis is the existence of potential coding errors though this likely would not occur differentially. In addition, patient data exploring psychosocial characteristics such as anxiety, depression, knowledge, beliefs, and barriers to treatment are not readily accessible. Future studies may explore these unmeasurable variables which contribute to survival. Fourth, this study only examined cancer patients with stage I–III tumors and thus the results are not generalizable to other stages of CRC. Fifth, given that this data only encompasses real‐world utilization data, it is not necessarily concordant with clinical practice guidelines. Further, it does allow for information on which patients may be ineligible for treatments such as chemotherapy. Finally, the findings of this study may not be generalizable to patients enrolled in insurance programs other than Medicare, under 65 years or with stage IV tumors. Accordingly, future studies may seek replication in more diverse study populations and time intervals.

## CONFLICT OF INTEREST

The authors declare that no competing interests exist

## Data Availability

The data that support the findings of this study are available from the Center for Medicaid and Medicare Services (CMS). Restrictions apply to the availability of these data, which were used under license for this study. Data used in the analyses are available from the authors with the permission of the CMS.
